# Comparison of self-ligating Damon3 and conventional MBT brackets regarding alignment efficiency and pain experience: A randomized clinical trial

**DOI:** 10.15171/joddd.2019.043

**Published:** 2019

**Authors:** Arezoo Jahanbin, Nadia Hasanzadeh, Sara Khaki, Hooman Shafaee

**Affiliations:** ^1^Dental Research Center, School of Dentistry, Mashhad University of Medical Sciences, Mashhad, Iran; ^2^Department of Orthodontics, School of Dentistry, Mashhad University of Medical Sciences, Mashhad, Iran

**Keywords:** Alignment, conventional brackets, Damon, orthodontic, pain, self-ligating

## Abstract

***Background.*** Self-ligating brackets might be more efficient than conventional appliance systems during the initial alignment stage of orthodontic treatment due to reduced frictional resistance. This study aimed to compare the alignment efficiency and pain experience of Damon3 self-ligating and MBT pre-adjusted brackets in the initial alignment stage.

***Methods.*** In this randomized clinical trial, 30 patients aged 14‒20 years, who needed non-extraction treatment in both maxillary and mandibular arches, were randomly assigned to two groups; 15 patients were treated with MBT pre-adjusted brackets, and 15 patients received Damon3 self-ligating brackets, both with 0.022-in slots. Alginate impressions were taken at the start of treatment (T0) and four monthly visits (T1, T2, T3, and T4). Little’s irregularity index (LII) was used to assess the tooth displacements. The patients rated their pain experience immediately after the insertion of the archwire, 4 hours, 24 hours, 3 days, 7 days, and at each monthly visit using a visual analog scale (VAS).

***Results.*** The rate of upper dental alignment between T0 and T4 was significantly higher with the Damon3 compared to MBT brackets (P=0.015). Although significantly more changes in the lower LII scores were observed during the first three months with the Damon3 system, the rate of improvement in the irregularity of lower teeth over the 4-month period was not significantly different between the two groups (P=0.50). The patients’ pain experience was not significantly different between the bracket groups (P=0.29).

***Conclusion.*** During the four-month alignment stage, significantly more improvement in the upper dental irregularity was observed with self-ligating compared to conventional brackets. The bracket type had no effect on pain experience during the alignment stage.

## Introduction

Self-ligating brackets (SLB) use an integrated system to enclose the bracket slot, assuming that eliminating ligating modules would, in turn, diminish friction, thus reducing treatment time.^1^

Self-ligating appliances have also been shown to provide a more reliable ligation, more efficient tooth movement, and better control of tooth rotation compared to conventional brackets. The self-ligating brackets have also resulted in a 4-month reduction in treatment time and four fewer sessions during treatment.^2^

Numerous in vitro studies have demonstrated very low friction in self-ligating systems.^3-5^ In general, laboratory findings do not accurately simulate in vivo conditions.^6^ It has been shown that resistance to sliding increased similarly in both self-ligating and conventional appliances by changing bracket angulation.^7^

Also, it seems that SLBs preserve anchorage better than CLBs (conventional-ligating brackets).^8^ SLB requires less chair time and chair side assistance because it eliminates the need for any ligation. Voudouris et al^9^ showed a four-fold faster archwire replacement with the use of self-ligating appliances.

It is well documented that one of the main side effects of fixed orthodontic treatment is pain or discomfort,^10,11^ which can dissuade the patient from treatment,^12^ lower patient’s cooperation,^13^ and compromise the results of orthodontic treatment.^14^ Different patient- and treatment-related factors can influence the experience of pain during orthodontic treatment. Patient-related factors, such as age, sex, and previous experiences of the patient, can affect pain sensation.^15^ One of the main treatment-related factors, especially during the initial alignment phase of treatment, is the amplitude of the force that archwires apply to the dentition. The quantity of the applied force and the amount of tooth movement are directly related to the frictional resistance between the archwire and bracket. The degree of resistance is directly related to the physical characteristics of the archwire, bracket material, archwire dimensions, and the type of archwire ligation.^15,16^ It has been shown that during aligning and sliding phases of treatment, SLB systems produce lower force levels, which, in turn, might reduce the pain and discomfort experienced by patients during orthodontic treatment.^15^

Heretofore, few clinical trials have compared the patient pain experience or the efficiency of alignment for self-ligating versus conventional appliances, and the results are controversial. This study aimed to compare the Damon3 MX and MBT pre-adjusted bracket systems regarding the alignment efficiency and the pain and discomfort experienced by patients during the initial stages of orthodontic treatment.

## Methods

Thirty subjects who met the inclusion criteria participated in this randomized clinical trial. The inclusion criteria consisted of females 14‒20 years of age, mild to moderate dental irregularity requiring non-extraction treatment, presence of all the permanent teeth at least up to the first molars, good oral hygiene, and periodontal health. Patients were excluded if they required orthognathic surgery to correct skeletal discrepancies, were taking medications, like NSAIDs or other anti-inflammatory drugs, had cleft lip or palate, hypodontia, or hyperdontia. Written consent forms were obtained after informing the patients and/or their parents of the interventions and the possible effects associated with them. Ethics approval for this clinical trial was obtained from the Ethics Committee under the code 1393.754.

Simple randomization without any stratification was carried out using the Excel computer program with a 1:1 allocation ratio. The participants were recalled from the waiting list, and each one was assigned a number. These assignments were hidden from the investigators using opaque letters until just before the placement of the appliance.

Both patient groups were treated with one orthodontist in a private office setting. In both groups, before bonding the brackets, the impressions for the maxillary and mandibular dentitions were taken using alginate impression material (T0). Self-ligating Damon 3MX or conventional MBT pre-adjusted brackets (Ormco Corporation, Orange, California, USA; 0.022-inch slot) were bonded on the teeth for the upper and lower arches at the same visit. An 0.014-inch round Cupper NiTi archwire (Ormco, California, USA) was first used for the alignment, followed by an 0.016-inch Cu NiTi wire at the second visit in both groups. The conventional MBT brackets were fully ligated using elastomeric ligatures.

No auxiliaries, such as lingual arches, bite plates or intermaxillary elastics, were used during the study period. During four months of study, the patients were recalled at the end of each month, and impressions of both arches were taken using alginate impression material (T1, T2, T3, and T4).

Little’s irregularity index^17^ was used to assess the changes in dental alignment throughout the study. All the measurements were made on the study models taken at T0, T1, T2, T3, and T4 using an electronic digital caliper (Dentaurum, Inspringen, Germany) with an accuracy of 0.01 mm. Intra-examiner reliability was assessed by randomly re-measuring five pairs of dental casts after four weeks by the same examiner. The intra-class correlation coefficient (ICC) was estimated at 0.92, indicating high intra-examiner reliability for the measurements.

The patients were asked to record their pain experience immediately after wire insertion, at 4 hours, 24 hours, 3 days, 7 days, and immediately after each monthly visit, using a 10-cm-long visual analog scale (VAS) in which the left end represented “no pain” at all and the right end signified the “worst pain imaginable” (one pain score for both arches). The patients were advised not to use any pain killers.

### Statistical analysis

Student’s t-test and repeated-measurements analysis, with the significance level of 0.05, were used to analyze the outcome measurements. SPSS 16.0 (IBM Corp., Armonk, New York, USA) was used to perform the analyses

## Results

Thirty female patients with 15 patients in each group were included in this study. Table 1 presents the mean age and mean pretreatment LII (Little's irregularity index) scores of the upper and lower dental arches for both groups. Independent-samples t-test showed no significant differences between the conventional MBT and Damon3 MX bracket groups in terms of age (P=0.78) and pretreatment lower LII scores (P=0.68). The pretreatment irregularity in the upper arch was significantly different between the two groups (P=0.03; Table 1).

**Figure 1:**
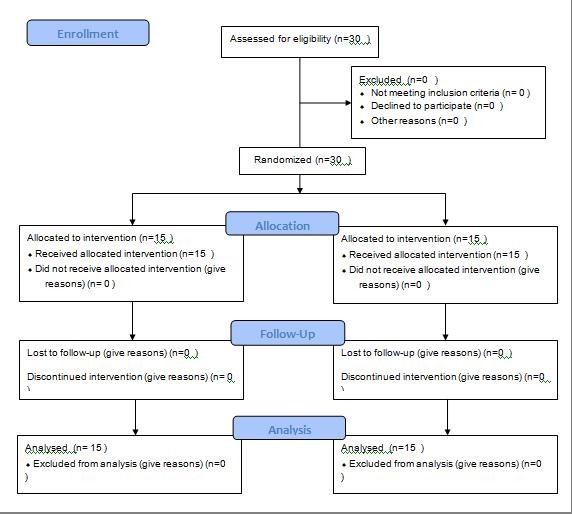
Consort flow diagram.

**Figure 2:**
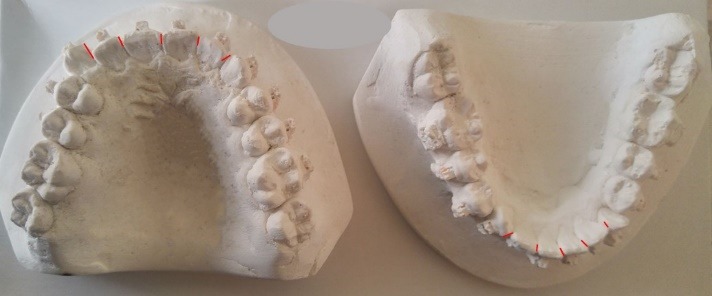
Assessment of Little’s irregularity index.

**Table 1:** Comparison of age distribution and pretreatment Little' irregularity index (LII) between the Damon3 MX and conventional MBT bracket groups

**Bracket type**	**Mean age (SD)**	**Mean pretreatment upper LII score (SD)**	**Mean pretreatment lower LII score (SD)**
**MBT (n=15)**	16.13 (1.25)	5.63 (1.45)	5.54 (2.41)
**Damon3 MX (n=15)**	16.26 (1.39)	6.90 (1.66)	4.67 (2.06)
**Comparison between groups**	T= -0.27 P=0.78	T= -2.23 P=0.03*	T=1.06 P=0.68

*P<0.05.

A comparison of the difference in the upper LII scores at sequential time points between self-ligating and conventional appliances are presented in Table 2. There was a statistically significant difference in the rate of upper tooth movement at T0-T1 between conventional MBT and Damon3 MX bracket types (P=0.02; Table 2). The rate of tooth movement during the 2nd, 3rd and 4th month of treatment was not significantly different between the two groups (Table 2).

**Table 2:** Comparison between the Damon3 MX and conventional MBT groups in the upper Little's irregularity index (LII) scores at the 4 treatment intervals: T0 = pretreatment and at the first (T1), second (T2), third (T3) and fourth (T4) month

	**Bracket type**	**Mean upper LII scores (SD)**	**Comparison between groups**	
			**T statistics**	**P-value**
**T0-T1 difference**	**MBT**	2.01 (0.62)	2.30	0.02*
	**Damon3 MX**	2.62 (0.83)		
**T1-T2 difference**	**MBT**	1.36 (0.94)	1.15	0.25
	**Damon3 MX**	1.71 (0.67)		
**T2-T3 difference**	**MBT**	1.01 (0.62)	0.24	0.81
	**Damon3 MX**	1.06 (0.64)		
**T3-T4 difference**	**MBT**	0.57 (0.74)	0.30	0.75
	**Damon3 MX**	0.64 (0.53)		

*P<0.05.

Comparison of the difference in overall upper dental alignment between T0 and T4 showed significantly more changes with Damon3 MX brackets compared to conventional MBT system over the 4-month alignment stage (P=0.015; Table 3).

**Table 3:** Comparison of the difference in the upper LII scores between T0 (pretreatment) and T4 (after alignment) for MBT and Damon3 MX bracket systems

**Bracket type**	**Mean T0 (SD)**	**Mean T4 (SD)**	**Mean T0-T4 (SD)**
**MBT**	5.63 (1.45)	0.68 (0.85)	4.95 (1.30)
**Damon3 MX**	6.90 (1.66)	0.86 (1.18)	6.04 (0.99)
**Comparison between groups**	T=2.23 P=0.034*	T=0.47 P=0.63	T=2.58 P=0.015*

*P<0.05.

Table 4 demonstrates the difference in the lower LII score changes during treatment between the two groups. The rate of tooth movement in the lower arch during the 1st, 2nd, and 3rd months of treatment was significantly different between the conventional MBT and Damon3 MX bracket types (Table 4). However, as shown in Table 5, the change in lower LII score between T0 and T4 (overall tooth alignment) was not significantly different between Damon3 MX and MBT bracket groups (P=0.50).

**Table 4:** Comparison between the Damon3 MX and conventional MBT groups in the lower Little's irregularity index (LII) scores at the four treatment intervals: T0 = pretreatment and at the first (T1), second (T2), third (T3) and fourth (T4) months

	**Bracket type**	**Mean lower LII scores (SD)**	**Comparison between groups**	
			**T statistics**	**P-value**
**T0-T1 difference**	**MBT**	1.55 (0.76)	-2.86	0.009*
	**Damon3 MX**	2.66 (1.30)		
**T1-T2 difference**	**MBT**	1.57 (0.73)	2.69	0.012*
	**Damon3 MX**	0.87 (0.69)		
**T2-T3 difference**	**MBT**	1.06 (0.81)	2.89	0.008*
	**Damon3 MX**	0.36 (0.48)		
**T3-T4 difference**	**MBT**	0.55 (0.45)	1.38	0.177
	**Damon3 MX**	0.37 (0.22)		

*P<0.05.

**Table 5:** Comparison of the difference in the lower LII scores between T0 (pretreatment) and T4 (after alignment) for MBT and Damon3 MX bracket systems

**Bracket type**	**Mean T0 (SD)**	**Mean T4 (SD)**	**Mean T0-T4 (SD)**
**MBT**	5.54 (2.41)	0.82 (0.70)	4.27 (0.47)
**Damon3 MX**	4.67 (2.06)	0.41 (0.44)	4.26 (1.95)
**Comparison between groups**	T=1.06 P=0.29	T=1.88 P=0.06	T=0.67 P=0.50

Mean pain scores of patients in each group at nine time intervals during treatment are presented in Table 6. The level of patients’ pain and discomfort significantly decreased during the sequential time intervals of treatment in each group (P<0.001), but the bracket type had no influence on pain experience during the alignment stage (P=0.29; Table 6).

**Table 6:** Mean pain scores of patients at different time intervals using VAS data in the two groups

**Time point**	**Bracket type**	**Mean pain scores (SD)**
**After archwire insertion**	MBT	5.67 (1.29)
	Damon3 MX	7.27 (1.33)
**4 hours**	MBT	4.20 (1.37)
	Damon3 MX	4.33 (1.91)
**1 day**	MBT	4.60 (1.18)
	Damon3 MX	4.13 (1.24)
**3 days**	MBT	3.20 (0.94)
	Damon3 MX	2.97 (1.04)
**7 days**	MBT	1.20 (0.68)
	Damon3 MX	1.41 (0.91)
**1st month**	MBT	0.33 (0.49)
	Damon3 MX	0.47 (0.52)
**2nd month**	MBT	0.40 (0.51)
	Damon3 MX	0.40 (0.51)
**3rd month**	MBT	0.27 (0.46)
	Damon3 MX	0.33 (0.49)
**4th month**	MBT	0.20 (0.41)
	Damon3 MX	0.27 (0.46)
**Repeated-measurements analysis**	Effect of time: F= 137.93, P<0.001 Effect of bracket type: F=1.16, P=0.29	

## Discussion

It has been assumed that very low frictional force with various designs of self-ligating brackets results in a faster alignment of the dentition and, in turn, lowers the overall treatment time.^18^ In particular, the Damon system has claimed that it presents advantages over conventional and other self-ligating systems for both the orthodontists and patients.^19^ The results of this randomized clinical study showed that over a 4-month treatment period, alignment of the upper teeth was significantly faster with self-ligating Damon3 MX system compared to conventional MBT pre-adjusted brackets, which might be due to the significant differences between the groups. Compared to the assumed level of clinical significance in incisor irregularity (2 mm), the observed difference in the upper mean T0-T4 between the two groups is unlikely to be clinically significant. Although significantly lower LII score changes were observed during the first three months of alignment with Damon3 MX compared to conventional MBT brackets, the rate of improvement in lower teeth irregularity over the 4-month period was not significantly different between the two bracket groups. This finding corroborates the results of a clinical trial by Ribeiro et al.^20^ The authors found no statistically significant differences between self-ligating and conventional bracket systems in the correction of lower dental crowding during the initial alignment phase (after 180 days). On the other hand, after 600 days of treatment, the difference in the correction of mandibular crowding between the conventional and self-ligating groups was statistically significant.^20^

In an investigation with a split-mouth design by Miles et al,^21^ 60 patients were enrolled to compare the efficacy of Damon2 brackets and conventional twin brackets during the initial alignment of mandibular teeth. At both 10 weeks and 20 weeks after the start of treatment, the conventional twin bracket system showed a lower irregularity index score than Damon2 bracket by 0.2 mm, which was not clinically significant. The authors concluded that there was no significant difference between the conventional twin and the Damon2 bracket systems regarding alignment efficiency during the initial stages of orthodontic treatment.^21^ In another study by Miles et al with a similar study design,^22^ the Smart clip self-ligating bracket was not more effective in reducing lower dental irregularity compared to a conventional twin bracket ligated with stainless steel ligatures or elastomeric modules during the initial stages of treatment. These findings are comparable to our results regarding the resolution of mandibular crowding. Likewise, in an investigation by Scott et al,^23^ no significant difference was noted in the initial rate of lower dental alignment for Damon3 self-ligating versus Synthesis conventionally ligated brackets.

Instead of comparing the improvements in LII during a specific period, Pandis et al decided to calculate the time needed to correct the lower anterior teeth.^24^ They investigated 54 subjects with mandibular irregularity index of >2, who needed non-extraction treatment to compare the efficacy of Damon2 self-ligating brackets with Microarch conventional edgewise appliance. The authors concluded that, in general, there was no difference between Damon2 and conventional brackets regarding the time needed to correct mandibular crowding during the initial alignment stage.^24^ However, for moderate mandibular crowding (LII<5 mm), 2.7 times faster correction was observed with the Damon2 bracket system. For greater crowding (LII>5 mm), the treatment time increased by an additional 20% for each irregularity index, regardless of bracket type.^24^

In cases of severe dental crowding and rotations, a full closure of the sliding cap in self-ligating appliances might not be possible because of excessive archwire bending. Failure of full archwire engagement results in a lack of free sliding of archwire within the bracket slot, which might significantly lower the rate of tooth movement in severely crowded cases.

Most previous clinical trials on the efficacy of self-ligating brackets during the alignment stage only considered the lower dental irregularity, not the upper crowding. In the present study, we found a significantly faster correction of upper dental irregularities with self-ligating Damon3 MX system compared to MBT pre-adjusted brackets over the 4-month alignment stage. The difference observed in the results of our study regarding the correction rate of mandibular and maxillary crowding might be related to the lower trabecular density of maxillary alveolar bone, which facilitates orthodontic tooth movement.

Some previous works conducted by Eberting^1^ and Harradine et al^8^ showed significantly lower treatment and fewer treatment sessions using Damon brackets versus conventionally ligated edgewise brackets. However, because of the retrospective designs of these studies, there is some potential risk for bias. Furthermore, it seems that any decrease in treatment time with SLBs might happen during the later stages of treatment, such as space closure in extraction cases, which does not happen during the initial alignment phase. The retrospective studies discussed earlier included both extraction and non-extraction patients (40% extraction rate in the study by Harradine et al^2^) that reflects a more complex series of cases. In addition, the matching method of subjects in the study groups and the sequence of archwires used were not mentioned in those investigations.

In the present study, the level of pain and discomfort experienced at different time intervals during the initial alignment stage was not significantly different between Damon3 MX and conventional MBT bracket groups. Similarly, in a systematic review by Celar et al,^25^ no significant difference in initial pain was found between self-ligating and conventional brackets. Likewise, in the study conducted by Fleming et al,^26^ the subjective pain experienced during the first week of fixed orthodontic treatment was not influenced by the bracket type (self-ligating SmartClip versus conventional Victory). These findings are consistent with the study of Scott et al,^27^ which found no significant differences in patients’ pain and discomfort between Damon3 self-ligating and conventional pre-adjusted brackets.

On the other hand, in the study conducted by Miles et al,^21^ patients in the conventional twin bracket group reported a significantly higher discomfort than the Damon 2 group during the first few days of alignment phase using 0.014-inch NiTi archwire. However, when the 0.016*0.025-inch archwire was used, Damon2 self-ligating group reported significantly higher discomfort than the conventional bracket group. The authors of this split-mouth-design study stated that the 0.028-inch depth for the bracket slot of Damon 2 appliance allowed an 8.5º rotational play with an 0.014-inch archwire and thus caused less pain and discomfort compared to the conventional system with no play at the bracket slot. At the insertion of the second 0.016*0.025-inch archwire, the average lower incisor irregularity corrected on the Damon2 side of the archwire was lower than the conventional bracket side, which resulted in more pain experience with Damon2 self-ligating brackets.^21^ In a split-mouth design, the dentition’s response to different bracket systems could be directly compared for each person, and the levels of discomfort could also be personally compared between these appliances without a need for VAS. However, in the present study, we did not apply a split-mouth design since using conventional brackets, and elastomeric modules on half of the arch inhibit the free sliding of wire in the Damon brackets past the midline, affecting the efficacy of self-ligating brackets. Furthermore, patients may not correctly specify the source of pain in areas close to the midline.

The rate of bracket debonding and breakage also affects the efficiency of treatment. A higher rate of bracket failure can be harmful to the office’s reputation, increases the treatment time, and requires additional clinical time for the repair and rebonding of the brackets. In some previous studies, a higher failure rate of the Damon system has been reported, which could be due to the clinician’s inexperience with the bracket system and the shearing force that is applied when opening or closing the slides of the brackets.^22^ In the current study, the rate of bracket breakage was not recorded. Moreover, another limitation of this study is that arch length and inter-canine and inter-molar widths were not measured at the beginning or after alignment to determine whether dental alignment was due to the proclination or mesiodistal movement of the teeth. Further clinical trials on the efficacy of Damon brackets with larger sample sizes, longer treatment periods, and inclusion criteria of extraction patients are recommended.

## Conclusion

The results of the present study indicated that over a 4-month alignment stage, more correction of the upper crowding occurred with the self-ligating Damon3 MX system compared with conventional MBT brackets. Regarding the basic differences in the upper dental crowding between the two groups, these results should be interpreted with caution. Although significantly more correction of lower irregularities occurred with Damon3 MX brackets during the first three months, the overall rate of dental alignment in the lower arch was not significantly different between the two groups. The type of bracket system resulted in no significant effect on subjective pain experience during the initial alignment stage of orthodontic treatment.

## Acknowledgements

This study was supported by a grant from the Vice Chancellor of Research of Mashhad University of Medical Sciences. The results presented in this work have been taken from a postgraduate student’s thesis (thesis number: 608).

## Authors’ Contributions

AJ contributed to the design and implementation of the research, NH participated in planning the work and wrote the manuscript with input from all the authors. SKH performed the measurements, and HSH contributed to the interpretation of the results. All the authors discussed the results and commented on the manuscript.

## Funding

Not applicable.

## Competing interests

The authors declare no competing interests with regards to the authorship and/or publication of this article.

## Ethics Approval

Ethical approval for this research was obtained from the Ethics Committee of Mashhad University of Medical Sciences under the code 1393.754.
